# Tunable critical temperature for superconductivity in FeSe thin films by pulsed laser deposition

**DOI:** 10.1038/s41598-018-22291-z

**Published:** 2018-03-06

**Authors:** Zhongpei Feng, Jie Yuan, Ge He, Wei Hu, Zefeng Lin, Dong Li, Xingyu Jiang, Yulong Huang, Shunli Ni, Jun Li, Beiyi Zhu, Xiaoli Dong, Fang Zhou, Huabing Wang, Zhongxian Zhao, Kui Jin

**Affiliations:** 1 Beijing National Laboratory for Condensed Matter Physics, Institute of Physics, Chinese Academy of Sciences, Beijing, 100190 China; 2 Key Laboratory of Vacuum Physics, School of Physical Sciences, University of Chinese Academy of Sciences, Beijing, 100049 China; 3 Research Institute of Superconductor Electronics, Nanjing University, Nanjing, 210093 China; 4 Collaborative Innovation Center of Quantum Matter, Beijing, 100190 China

## Abstract

Stabilized FeSe thin films in ambient pressure with tunable superconducting critical temperature would be a promising candidate for superconducting electronic devices. By carefully controlling the depositions on twelve kinds of substrates using a pulsed laser deposition technique single crystalline FeSe thin films were fabricated. The high quality of the thin films was confirmed by X-ray diffraction with a full width at half maximum of 0.515° in the rocking curve and clear four-fold symmetry in *φ*-scan. The films have a maximum *T*_*c*_ ~ 15 K on the CaF_2_ substrate and were stable in ambient conditions air for more than half a year. Slightly tuning the stoichiometry of the FeSe targets, the superconducting critical temperature becomes adjustable below 15 K with quite narrow transition width less than 2 K. These FeSe thin films deposited on different substrates are optimized respectively. The *T*_c_ of these optimized films show a relation with the out-of-plane (*c*-axis) lattice parameter of the FeSe films.

## Introduction

The Fe-based superconductors have attracted a lot of attention due to their intriguing superconducting properties and their promise towards novel applications^1,2^. Among various Fe-based superconductors, the 11-system possesses the simplest structure as the anti-PbO-type structure (space group of *P*4*/nmm*)^3,4^ but displays the most multifarious physical properties in all Fe-based superconductors. The 11-system includes the FeSe_1±*x*_^5,6^, Fe_1±*y*_(Se,Te)^7,8^ and Fe_1±*y*_(Se,S)^9^, among which the FeSe deserves more attentions. The FeSe bulk crystals exhibit an onset superconducting critical temperature (*T*_*c*_) of 9 K in ambient pressure^5^, which can be enhanced up to 38 K under external pressure. This effect is attributed to a decrease of anion height from the Fe-square planes, highlighting the impact of crystal lattice on superconductivity^10–14^. Unexpectedly, the *T*_*c*_ can be enhanced to 65 K for one unit cell (UC) of FeSe on SrTiO_3_ substrate^15–21^. Because of the extreme sensitivity to oxygen, such ultra-thin films of one or a few UC can only be investigated by combining *in-situ* fabrication and characterization. This drastically limits the possibilities to conduct research or develop novel applications based on this intriguing material. Therefore, more stable FeSe films, comparable to or even better than the bulk crystals, are highly desired for the next generation of Fe-based superconducting electronic devices.

The fabrication of FeSe thin films has been widely studied using pulsed laser deposition (PLD)^2,6,22–27^ and molecular beam epitaxy (MBE)^2,15–21,28,29^. From application point of view, PLD is much more efficient for the growth of films with moderate thickness (above 100 nm). The FeSe can be grown on various substrates, such as LaAlO_3_, SrTiO_3_ and MgO, but the obtained *T*_*c*_ values are generally equal to or lower than the *T*_c_ of bulk crystals. A recent report showed that CaF_2_ substrates could enhance *T*_*c*_ up to 15 K for FeSe films with a thickness of 150 nm^27^. The strain, induced by mismatch between the substrate and the film, may play a role in promoting *T*_*c*_^25^. However, it usually takes effect within limited film thickness, plausibly for the ultra-thin FeSe films where the *T*_*c*_ decreases quickly from 1 UC to 3 UC^18^. As such, it is still an open question why the *T*_*c*_ is enhanced in these thick films. Besides strain^23,25,29^, other effects, such as modification of the out-of-plane lattice parameter^13^, sample inhomogeneity by Fe vacancies^30–33^, as well as the growth conditions^28^, could also influence the superconducting properties. To solve this intriguing scientific puzzle, it is important to systematically study the crystal lattice and superconducting properties of FeSe films on various substrates.

In this work, we report on the successful synthesis of a series of high-quality single crystalline superconducting FeSe films on various substrates by PLD, with a maximum *T*_*c*_ up to 15 K. Besides, different growth parameters such as the ratio of Fe to Se in the targets and the thickness of the films are also elaborately tuned to arrive at a broad range of *T*_*c*_. Based on abundant high quality and stabilized samples, the relation between the crystal lattice and the tunable *T*_*c*_ has been carefully studied.

## Results

**Figure 1:**
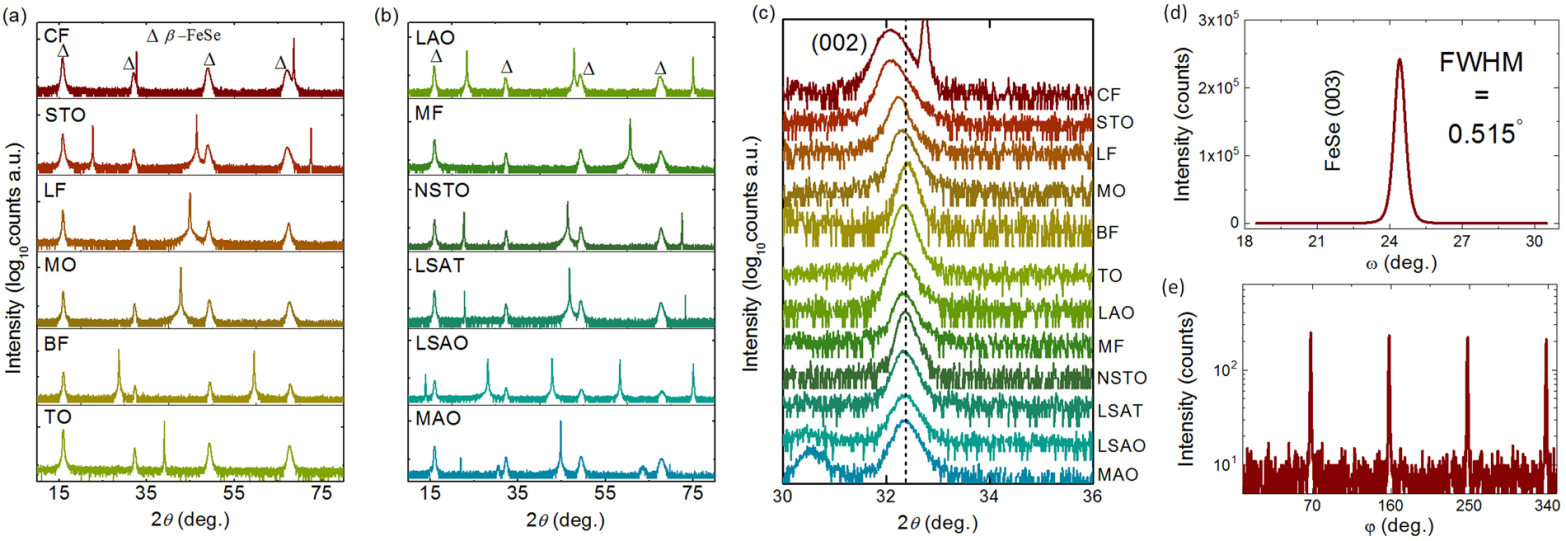
X-ray diffraction data of FeSe thin films. (**a**,**b**) The XRD *θ*-2*θ* scan data for FeSe thin films on various substrates. The triangles mark the Bragg diffraction peaks. The order is CaF_2_ (CF) SrTiO_3_ (STO), LiF (LF), MgO (MO), BaF_2_ (BF), TiO_2_ (100) (TO), LaAlO_3_ (LAO), MgF_2_ (MF), Nb-doped SrTiO_3_ (NSTO), La_0.3_Sr_0.7_Al_0.65_Ta_0.35_O_3_ (LSAT), (Sr,La)AlO_4_ (SLAO), and MgAl_2_O_4_ (MAO). (**c**) The enlarged view of the FeSe(002) peaks. These peaks exhibit obvious shift for different substrates. Here, the dash line is related to the (002) peak of FeSe/MAO. (**d**) The XRD rocking curve data of FeSe/CaF_2_. (**e**) The XRD *φ*-scan data of FeSe/CaF_2_. A four-fold symmetry of FeSe(011) diffraction peak indicates a high-quality epitaxial growth.

First, all the films on various substrates were grown with the same thickness of ~160 nm for a better comparison, as well as to release the epitaxial strain. The twelve substrates, explored in this work, are CaF_2_ (CF), SrTiO_3_ (STO), LiF (LF), MgO (MO), BaF_2_ (BF), TiO_2_(100) (TO), LaAlO_3_ (LAO), MgF_2_ (MF), Nb-doped SrTiO_3_ (NSTO), La_0.3_Sr_0.7_Al_0.65_Ta_0.35_O_3_ (LSAT), (Sr,La)AlO_4_ (SLAO), and MgAl_2_O_4_ (MAO). The material information of these substrates is summarized in Table 1. Figure 1(a,b) show the X-ray diffraction (XRD) data for the FeSe thin films grown on these substrates. All XRD *θ*-2*θ* scan patterns show a high quality (00 *l*) orientated growth. The measured (002) peaks, shown in Fig. 1(c) and plotted as a function of the measured critical temperature, display a slightly leftward shift with increasing *T*_c_. The *c*-axis lattice parameters were calculated from XRD *θ*-2*θ* scan data by the Bragg’s law, and the results will be discussed in the latter part. The full width at half maximum (FWHM) of the XRD rocking curve is 0.515*°*, showing high crystalline quality. In addition, the high quality epitaxy of the different films is confirmed by a clear four-fold symmetry in the XRD *φ* scan pattern for the (011) diffraction peak, as shown in Fig. 1(e).

**Table 1:** Structure parameters of various substrates.

Substrate	Crystal Plane	Atomic Distance at Surface (Å)	Mismatch (%)	*T*_*c*_-onset (K)
CaF_2_	(0 0 1)	*a*_0_ × √2/2 = 3.8679	2.60	15.17
LiF	(0 0 1)	*a*_0_ = *b*_0_ = 4.0270	6.82	14.01
SrTiO_3_	(0 0 1)	*a*_0_ = *b*_0_ = 3.9050	3.58	11.99
MgO	(0 0 1)	*a*_0_ = *b*_0_ = 4.2110	11.70	9.23
BaF_2_	(0 0 1)	*a*_0_ × √2/2 = 4.6810	24.16	9.24
TiO_2_	(1 0 0)	*b*_0_ = 4.2110, *c*_0_ = 2.9580	21.83, 21.54	7.86
LaAlO_3_	(0 0 1)	*a*_0_* = b*_0_ = 3.720	0.58	6.41
MgF_2_	(0 0 1)	*a*_0_ = *b*_0_ = 4.6200	22.55	5.51
Nb:SrTiO_3_	(0 0 1)	*a*_0_ = *b*_0_ = 3.9050	3.58	5.20
LSAT	(0 0 1)	*a*_0_ = *b*_0_ = 3.8680	2.60	4.75
(La,Sr)AlO_3_	(0 0 1)	*a*_0_ = *b*_0_ = 3.7560	0.37	6.93
MgAl_2_O_4_	(0 0 1)	*a*_0_ = *b*_0_ = 8.0830	7.20	5.96

**Figure 2:**
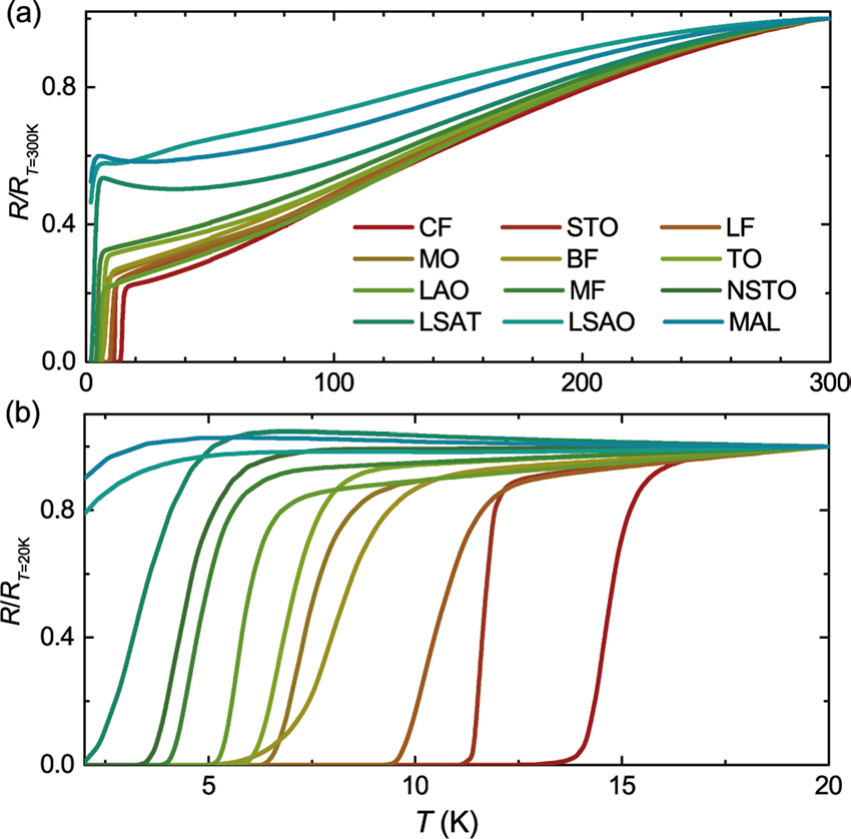
Temperature dependence of normalized resistance *R/R*_*N*_ for FeSe thin films with respect to various substrates. Here, *R*_*N*_ corresponds to the resistance at 300 K and 20 K for (**a**) and (**b**), respectively, and the thickness of all films are ~160 nm.

Figure 2 shows the temperature dependence of the resistance (*R-T*) curves for all fabricated films. Since the fabrication process is identical, the superconducting properties strongly depend on the substrates. In most samples, a zero-resistance transition can be observed except the films on the substrates of MAO and LSAO, for which a low-*T* upturn occurs in the *R-T* curves above 2 K. Comparing the films deposited on all substrates, the FeSe/CF films are selected for further exploration, since the *T*_*c*_ is the highest in this work.

**Figure 3:**
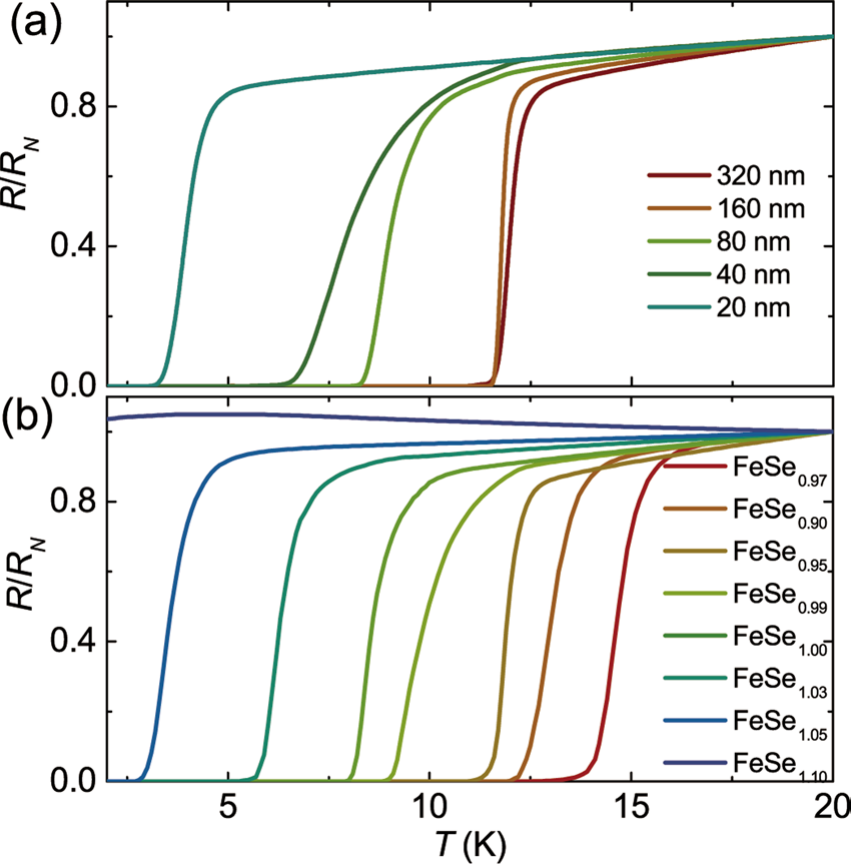
The normalized resistance vs. temperature (*R*/*R*_*N*_-*T*) curves for FeSe/CaF_2_ thin films with respect to thicknesses and targets. Here, the *R*_*N*_ was defined as the resistance at 20 K. (**a**) A series of FeSe/CaF_2_ films with various thicknesses are fabricated by only adjusting the laser pulsed counts. Here, all films were deposited at 350 °C by using the same target (Fe:Se = 1:0.95). (**b**) The FeSe/CaF_2_ films are grown from different targets in a range of Fe:Se ratio from 1:1.10 to 1:0.90, in which the film deposited from the Fe:Se = 1:0.97 target is observed the highest *T*_*c*_. The order of films fabricated by different FeSe_1±*x*_ targets is arranged along *T*_*c*_ increasing, namely, from FeSe_1.10_ to FeSe_1.05_, FeSe_1.03_, FeSe_1.00_, FeSe_0.99_, FeSe_0.95_, FeSe_0.90_, and FeSe_0.97_.

Since the thickness is considered as a key factor for superconductivity of thin films^22,23,25,29^, especially the ultra-thin FeSe system^15,18,29^, we also studied the thickness dependence of *T*_*c*_ for the FeSe/CF films. The films with different thicknesses were prepared by only adjusting the pulsed laser counts, while keeping the same FeSe_0.95_ target. In Fig. 3(a), we show the *R-T* curves for the FeSe films with different thicknesses. Superconductivity still remains as the thickness is reduced to 20 nm and 10 nm (about 36 and 18 UC) respectively, where *T*_*c*0_ = 3.4 K and 2 K, indicating the high quality of our FeSe films with less disorder and the stability of our PLD chamber. Increasing film thickness, the *T*_*c*_ also ascends gradually and the films almost display a bulk-like behavior when the thickness exceeds 160 nm.

In previous work, the superconductivity of FeSe films is found to be extremely sensitive to the stoichiometry between Fe and Se^30^. Therefore, considering the composition off-stoichiometry for the superconductivity of FeSe films, namely the vacancies or interstitial impurity^30,32^, we prepared the targets with subtle adjustment in the nominal Fe:Se, including 1:1.10, 1:1.05, 1:1.03, 1:1.00, 1:0.99, 1:0.97, 1:0.95, 1:0.90, and so on. For comparison, the films were grown with the same thickness of ~160 nm and on the same CF substrate. Figure 3(b) shows the *R-T* curves for films grown from different targets. The *T*_*c*_ of the films can be well tuned from < 2 K to 15 K with a pretty narrow transition width (Δ*T < *2 K). It should be noted that the best sample deposited by the FeSe_0.97_ target shows that Δ*T = *1.2 K, RRR = 5, *T*_*c*_* = *15 K, which can hold for more than half a year. By precise adjustment of the nominal composition, it seems that the ratio of Fe:Se is closely related to the superconductivity of the deposited films. Both energy dispersive X-ray spectra (EDX) and inductively coupled plasma atomic emission spectroscopy (ICP-AES) have been used to check the chemical composition. However, the composition between different superconducting films cannot be clearly defined by these two methods. Thus, we prefer to study on the stoichiometry of Fe and Se on the target rather than on the FeSe thin films themselves.

## Discussion

**Figure 4:**
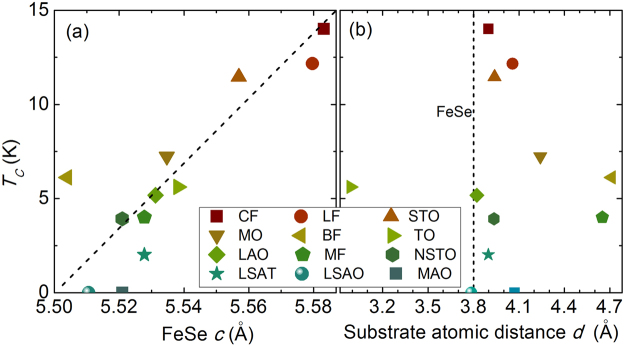
Lattice parameters dependence of superconductivity for FeSe thin films on various substrates. (**a**) *T*_*c*0_ versus *c* (FeSe lattice constant); (**b**) *T*_*c*0_ versus *d* (substrate atomic distance).

Figure 4(a) gives the *T*_*c*0_ (zero resistance superconducting critical temperature) with respect of the corresponding lattice constant *c* of FeSe films deposited on various substrates. The *T*_*c*0_ shows a clear positive correlation with the lattice constant *c* (the dashed line is a guide to the eye), rather than the in-plane lattice parameter (*d*) of the substrates (Fig. 4(b)), indicating the epitaxial strain from substrate has been released in films with thickness of 160 nm. In FeSe/TO films the substrate can induce an anisotropic epitaxial strain, because of the rectangular lattice on the surface of the TO substrate (*b = *4.593 Å, *c = *2.958 Å). However, the FeSe/TO films display a *T*_c0_ of 5.6 K, which is still comparable to that of certain films with isotropic strain. Therefore, the *c*-axis lattice constant is more closely related to the superconductivity of FeSe thin films. Comparably, the superconductivity of the multi-layered FeSe-based superconductors, i.e., (Li_1-*x*_Fe_*x*_)OHFeSe^34^ and Fe(Se_0.5_Te_0.5_)^35^, shows a similar *c*-axis constant dependence behavior, which reinforces our understanding on the profile of lattice parameters to the superconductivity. The *c*-axis lattice parameter depends on the anion height from the Fe layer^4,36,37^ (or the bond angle of Se-Fe-Se^4,38^) and the distance between two adjacent Fe_2_Se_2_ layer^39^, which will influence the magnetism and interlayer coupling respectively. Therefore, it deserves more in-depth study to uncover the internal mechanism for this positive correlation between *T*_*c*_ and lattice constant *c*.

In summary, we have successfully prepared high quality superconducting FeSe films on twelve different substrate type, such as CF, LF, STO, LAO, TO, MO, BF, MF, NSTO, LSAT, SLAO and MAO. Among them, the FeSe films deposited on CF_,_ LF and STO substrates possess the highest values of *T*_*c*_ for 15 K, 14 K and 12 K, respectively. By slightly adjusting the ratio of Fe to Se in the targets, a series of FeSe/CF films with tunable *T*_*c*_ values from < 2 K to 15 K are obtained. The superconductivity of the films (~160 nm) on various substrates is found to be mainly correlated with the *c*-axis lattice parameter of FeSe films. However, there is no direct correlation between the *T*_*c*_ and the surface atomic distance of substrates. The origin of the modification on *c*-axis parameter needs to be further identified, nevertheless, high-quality FeSe thin films with tunable *T*_*c*_ may pave the way for understanding the nature of FeSe from bulk crystal to ultrathin films, and shed light on the applications of superconducting microelectronic devices, such as the hybrid Josephson junctions, single-photon detection superconducting nanowires, and so on.

## Methods

FeSe polycrystalline targets were fabricated by the solid-state reaction method. The original materials of Fe (4 N, Alfa Aesar Inc.) and Se (5 N, Alfa Aesar Inc.) powders were mixed with designed ratio of stoichiometry, then heat-treated at 420 *°*C for 24 hours in evacuated quartz tubes. The as-prepared material was grinded and sintered at 450 *°*C for 48 hours, and such process was repeated more than three times for final targets. FeSe thin films were prepared by PLD technique with a KrF laser. The background vacuum of the deposition chamber is better than 10^*-*7^ Torr. The FeSe thin films were grown in vacuum with the target-substrate distance of ~ 50 mm, the laser energy of 350 mJ, the laser repetition of 2 Hz, and the substrate temperature of 350 °C. And then, the real deposition rate can be fixed at 1.3 nm/min.

X-ray diffraction (XRD) and X-ray reflection (XRR) measurements of the thin films were performed on a Rigaku SmartLab (9 kW) X-ray diffractometer with Ge(220) × 2 crystal monochromator. The Scanning Electron Microscope (SEM) measurements of the thin films were performed on a Hitachi Scanning electron microscope SU5000. In this work, for the thin films with thickness less than 80 nm, we check the thickness by both XRR and SEM. However, for the film thickness more than 80 nm, we will check them by SEM only. The transport properties of films were measured in the Physical Property Measurement System (PPMS-9 T).

## Electronic supplementary material

Supplementary Information
