# Supplementary material for: Tunable critical temperature for superconductivity in FeSe thin films by pulsed laser deposition
Source: Sci Rep. 2018 Mar 6;8:4039. doi: 10.1038/s41598-018-22291-z (PMC5840431; doi:10.1038/s41598-018-22291-z)
Supplement: Supplementary file 1 — Supplementary Information [file 41598_2018_22291_MOESM1_ESM.docx]

**Supplementary Information for “Tunable critical temperature for superconductivity in FeSe thin films by pulsed laser deposition”**

Zhongpei Feng,^1,2^ Jie Yuan,^1,2^ Ge He,^1,2^ Wei Hu,^1,2^ Zefeng Lin,^1,2^ Dong Li,^1,2^ Xingyu Jiang,^1,2^ Yulong Huang,^1,2^ Shunli Ni,^1,2^ Jun Li,^3^ Beiyi Zhu,^1^ Xiaoli Dong,^1,2^ Fang Zhou,^1,2^ Huabing Wang,^3^ Zhongxian Zhao,^1,2,4^ and Kui Jin^1,2,4^

*^1^ Beijing National Laboratory for Condensed Matter Physics, Institute of Physics, Chinese Academy of Sciences, Beijing 100190, China.*

*^2^ Key Laboratory of Vacuum Physics, School of Physical Sciences, University of Chinese Academy of Sciences, Beijing 100049, China*

*^3^ Research Institute of Superconductor Electronics, Nanjing University, Nanjing 210093, China*

*^4^ Collaborative Innovation Center of Quantum Matter, Beijing 100190, China.*


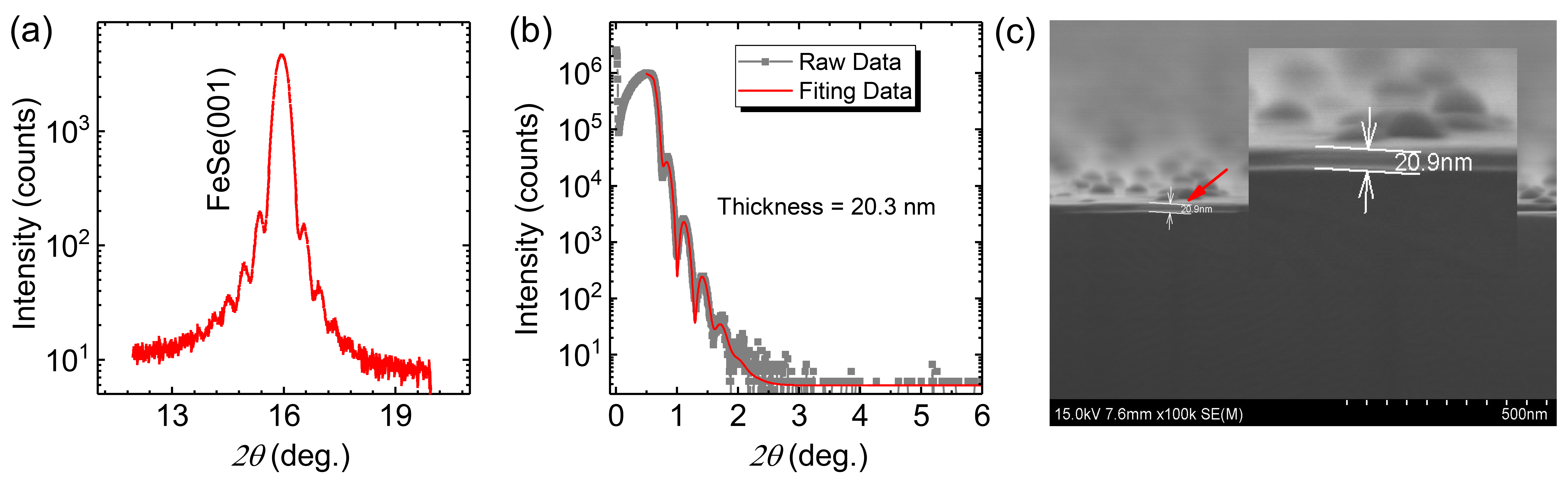


**Fig. S1.** (a) The X-ray diffraction θ-2θ scan data of FeSe thin films; (b) The X-ray reflection data of FeSe thin films; (c) The SEM image of the cross section for FeSe thin films. In the present work, for the thin films with thickness no more than 80 nm, we check the thickness by both XRR and SEM . However, for the film thickness larger than 80 nm, we will check them by SEM only.


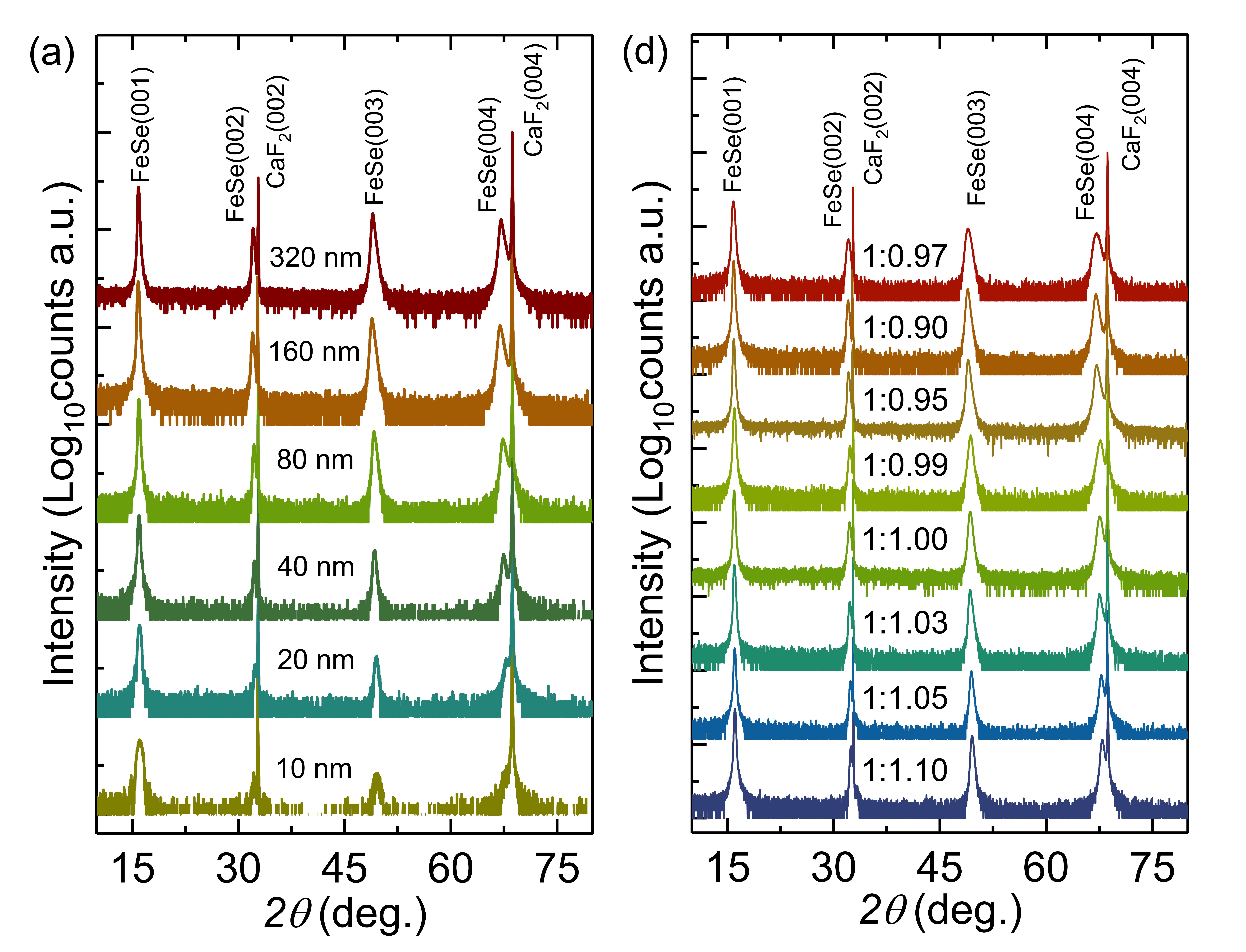


**Fig. S2.** (a), (b) The XRD data of FeSe/CaF_2_ films with various thicknesses and different target stoichiometry.


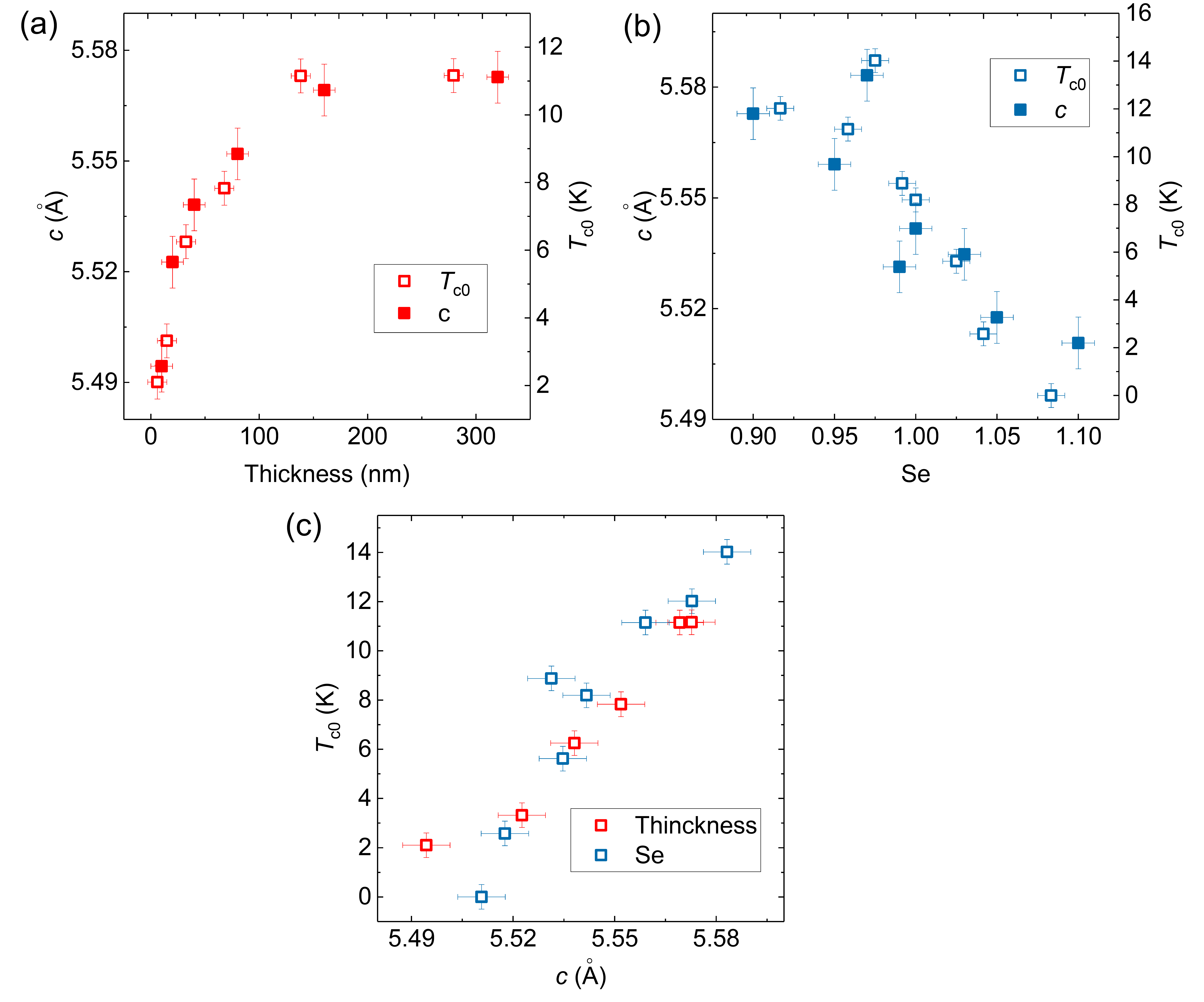


**Fig. S3.** (a) Thickness dependence of superconductivity and Lattice parameters for FeSe thin films; (b) The target Fe:Se ratio dependence of superconductivity and Lattice parameters for FeSe thin films; (c) *T*_c0_ versus *c* (FeSe lattice constant).
